# Supplementary material for: Effects of Prior Infection with SARS-CoV-2 on B Cell Receptor Repertoire Response during Vaccination
Source: Vaccines (Basel). 2022 Sep 6;10(9):1477. doi: 10.3390/vaccines10091477 (PMC9506540; doi:10.3390/vaccines10091477)
Supplement: Supplementary file 1 [file vaccines-10-01477-s001.zip › vaccines-1864207-supplementary.pdf]

SUPPLEMENTAL INFORMATION

Effects of prior infection with SARS-CoV-2 on B cell receptor repertoire response during vaccination.

Elizabeth R. Fraley, Santosh Khanal, Stephen H. Pierce, Cas A. LeMaster, Rebecca McLennan, Tomi Pastinen, and Todd Bradley

Table of contents

Figure S1: V gene usage, HCDR3 length distribution, and SHM of the IgM portion of the BCR repertoire. Page 2
Figure S2: V gene usage, HCDR3 length distribution, and SHM of the IgA portion of the BCR repertoire. Page 3
Figure S3. Distribution of most numerous clones by clonal frequency. Page 4
Figure S4: HCDR3 length distribution of top 50 IgG clones by serotype. Page 5
Table S1: Read depth and number of total clones. Page 6
Table S2: Top 50 clones by time point. Page 7

SUPPLEMENTAL FIGURES

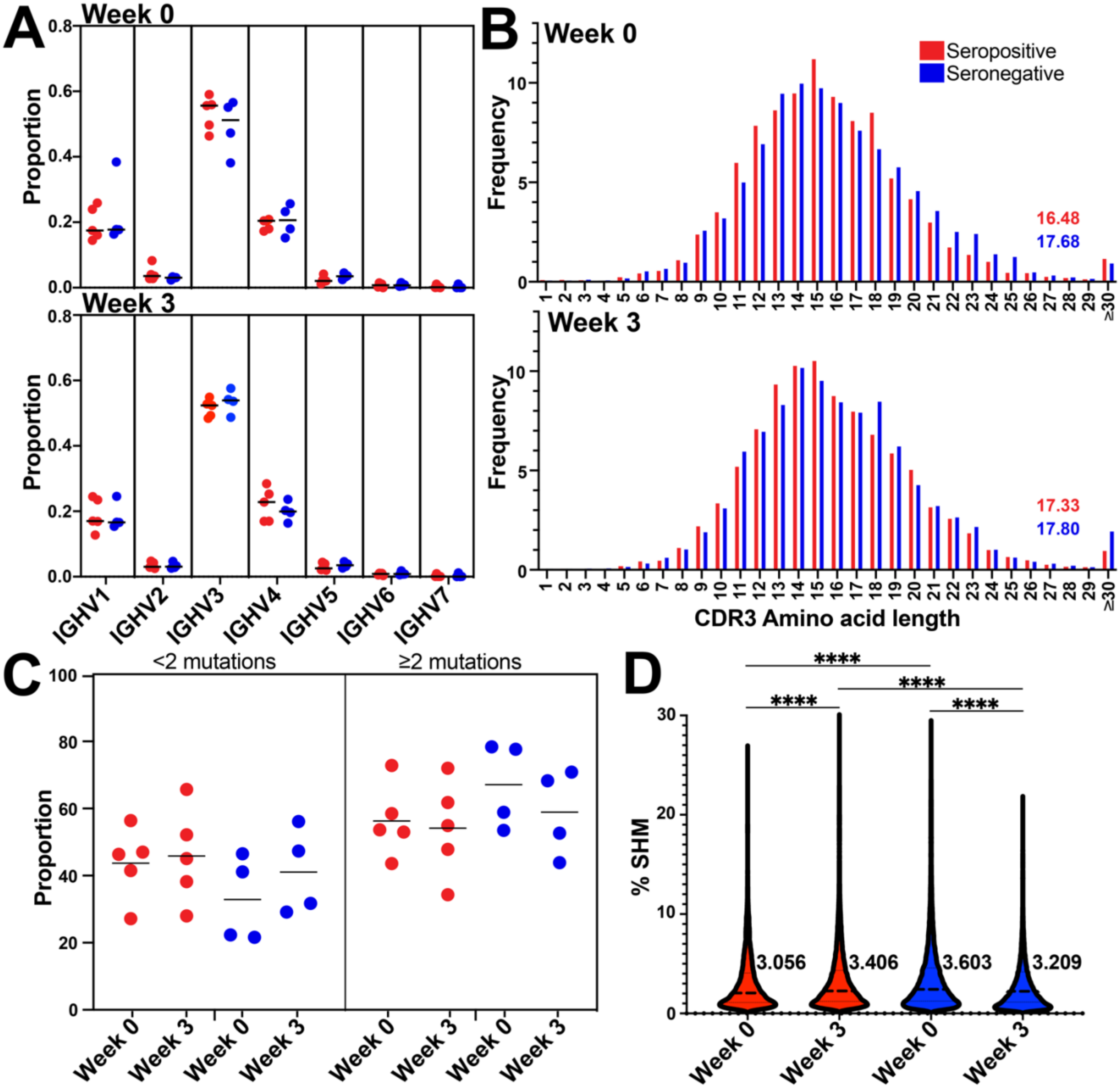

Figure S1: V gene usage, HCDR3 length distribution, and SHM of the IgM portion of the BCR repertoire. **(A)** Heavy chain V gene usage (IGHV1-7) of IgM clones. Proportion as indicated for seropositive (red) and seronegative (blue). No significant differences were observed between serotypes at week 0 and week 3, and no changes within serotype over time were observed. **(B)** Distribution of heavy chain complementary determining region 3 (HCDR3) lengths of IgM clones for week 0 and week 3. Seropositive (red) and seronegative (blue) and mean HCDR3 length are shown. No significant changes in the distribution of HCDR3 lengths were seen between serotypes at either time point, or within serotype between weeks. **(C)** Plot depicting the proportion (0-100%) of IgM clones with less than 2 mutations (left) and greater than 2 mutations (right). There were no statistically significant differences in the proportions of seropositive (red) and seronegative (blue) at week 0 or week 3. **(D)** Violin plots depicting percent of SHM (%SHM) for IgM clonotypes in seropositive (red) and seronegative (blue) at weeks 0 and 3. Mean values for each violin are indicated on the plot and by strong dashed lines, quartiles are indicated by thin dashed lines. Statistical test performed was Welch's t-test, p value **** $p<0.0001$.

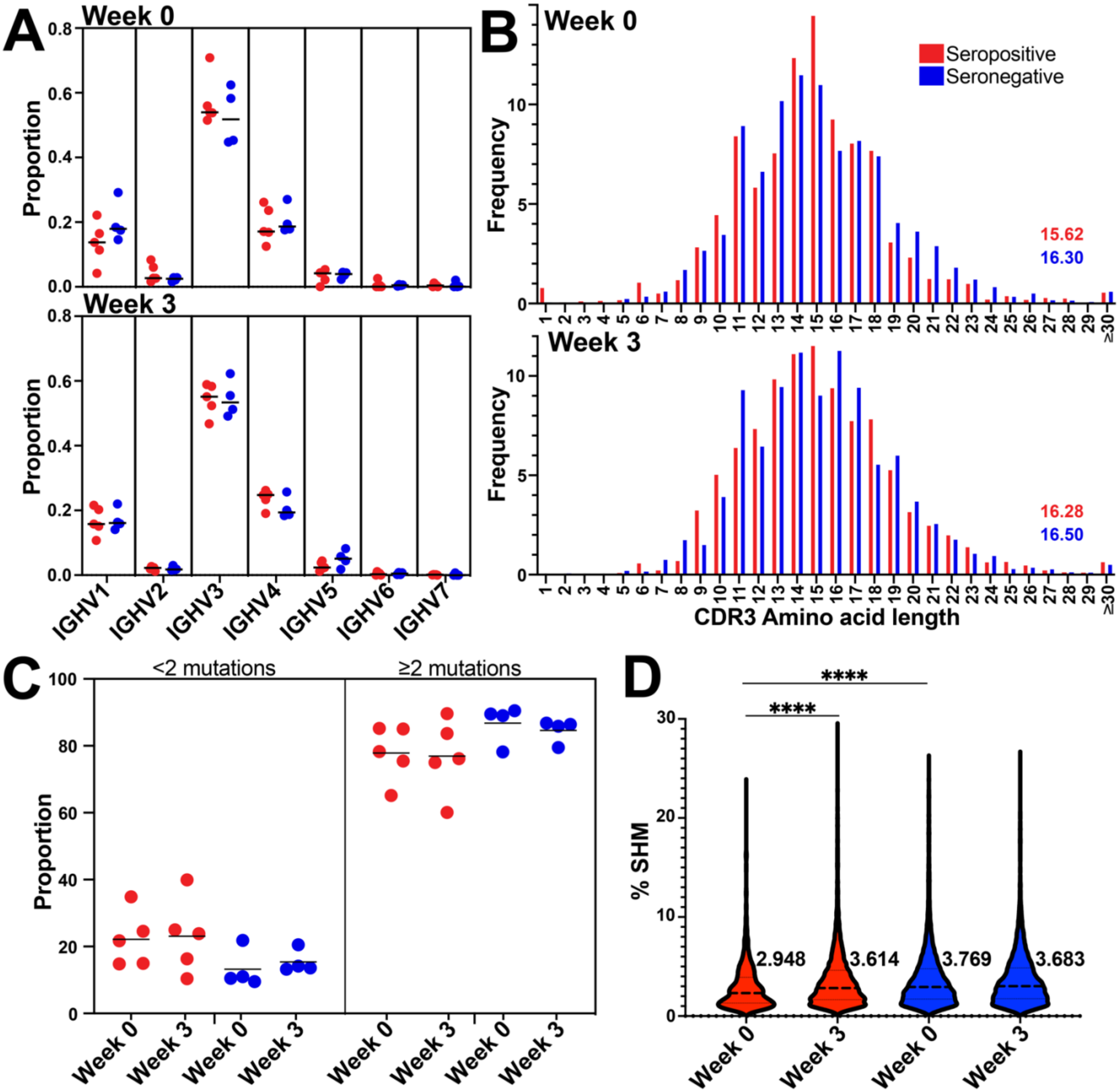

Figure S2: V gene usage, HCDR3 length distribution, and SHM of the IgA portion of the BCR repertoire **(A)** Heavy chain V gene usage (IGHV1-7) of IgA clones. Proportion as indicated for seropositive (red) and seronegative (blue). No significant differences were observed between serotypes at week 0 and week 3, and no changes within serotype over time were observed. **(B)** Distribution of heavy chain complementary determining region 3 (HCDR3) lengths of IgA clones for week 0 and week 3. Seropositive (red) and seronegative (blue) and mean HCDR3 length are shown. No significant changes in the distribution of HCDR3 lengths were seen between serotypes at either time point, or within serotype between weeks. **(C)** Plot depicting the proportion (0-100%) of IgA clones with less than 2 mutations (left) and greater than 2 mutations (right). There were no statistically significant differences in the proportions of seropositive (red) and seronegative (blue) at week 0 or week 3. **(D)** Violin plots depicting percent of somatic hypermutation (%SHM) for IgA clonotypes in seropositive (red) and seronegative (blue) at weeks 0 and 3. Mean values for each violin are indicated on the plot and by strong dashed lines, quartiles are indicated by thin dashed lines. Statistical test performed was Welch's t-test, p value **** $p<0.0001$.

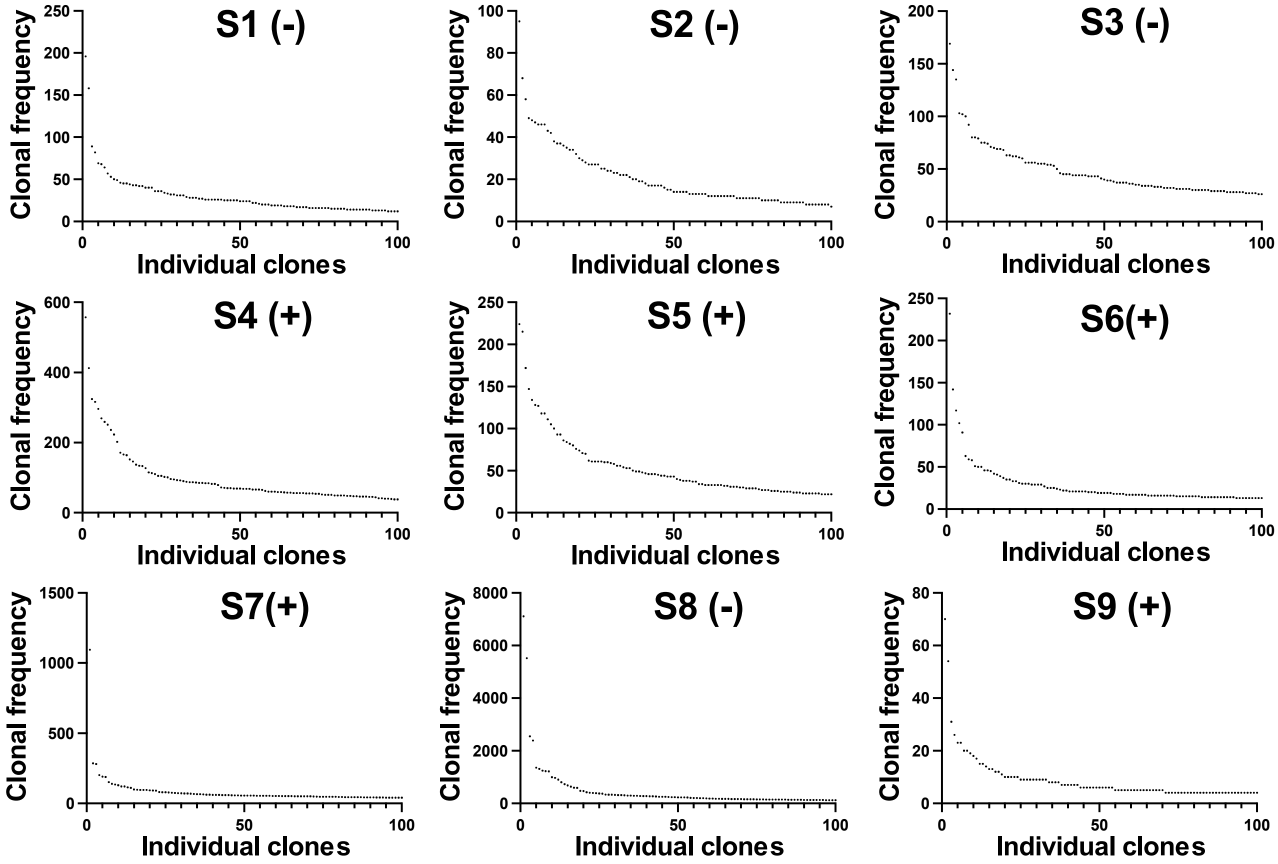

Figure S3. Distribution of most numerous clones by clonal frequency. Top 100 most numerous clones plotted in descending order by read number (clonal frequency) for each subject in this study (S1-9).

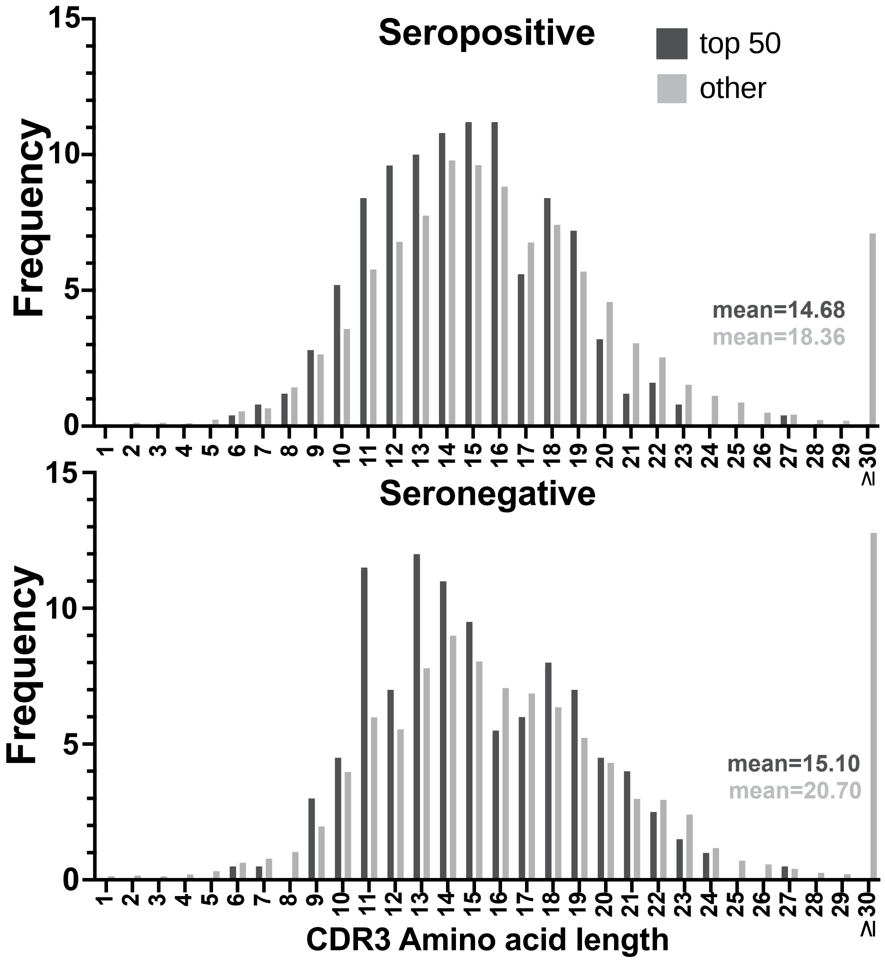

Figure S4: HCDR3 length distribution of top 50 IgG clones by serotype. HCDR3 length distribution of seropositive (top), and seronegative (bottom). Means are indicated for the top 50 (dark gray) and other (light gray). Kolmogorov-Smirnov test was performed, * p=0.0354).

SUPPLEMENTAL TABLES

Table S1. Read depth and number of total clones.

	# of reads pre-QC	# of reads post-QC	# of unique clones	Status	RNA conc. (ng)
sample 1, week 0	4320757	538707	66277	seronegative	786
sample 1, week 3	3031410	83141	9282	seronegative	1600
sample 2 week 0	1333079	83092	11085	seronegative	1600
sample 2 week 3	3055257	160469	24433	seronegative	1600
sample 3, week 0	1946747	70920	7995	seronegative	1600
sample 3, week 3	3784400	196798	19786	seronegative	1600
sample 4, week 0	4398520	75333	3975	seropositive	1112
sample 4, week 3	4,102,982	276158	26116	seropositive	1564
sample 5, week 0	863,397	75240	688	seropositive	1516
sample 5, week 3	3,095,188	207393	26385	seropositive	1600
sample 6, week 0	3537122	104344	10660	seropositive	1600
sample 6, week 3	3634992	333502	43888	seropositive	1600
sample 7, week 0	4451395	194339	9409	seropositive	1600
sample 7, week 3	2,230,122	461031	58672	seropositive	1600
sample 8, week 0	6001878	347447	24080	seronegative	1204
sample 8, week 3	3066931	447007	25337	seronegative	1150
sample 9, week 0	940760	222153	32910	seropositive	1600
sample 9, week 3	1012370	54974	8140	seropositive	1600

Supplemental Table S2. Number of expanded clones “top 50” present at each time point and percentage of overlap with other time points by sample.

sample	# of clones	percentage	group		
sample 1	13	26	top 50 at time point 1 present at time point 2		
-	4	8	top 50 at time point 1 present at top 50 time point 2		
	14	28	top 50 at time point 2 present at time point 1		
sample 2	6	12	top 50 at time point 1 present at time point 2		
-	1	2	top 50 at time point 1 present at top 50 time point 2		
	6	12	top 50 at time point 2 present at time point 1		
sample 3	12	24	top 50 at time point 1 present at time point 2		
-	2	4	top 50 at time point 1 present at top 50 time point 2		
	5	10	top 50 at time point 2 present at time point 1		
sample 4	11	22	top 50 at time point 1 present at time point 2		
+	1	2	top 50 at time point 1 present at top 50 time point 2		
	2	4	top 50 at time point 2 present at time point 1		
sample 5	17	34	top 50 at time point 1 present at time point 2		
+	0	0	top 50 at time point 1 present at top 50 time point 2		
	0	0	top 50 at time point 2 present at time point 1		
sample 6	14	28	top 50 at time point 1 present at time point 2		
+	2	4	top 50 at time point 1 present at top 50 time point 2		
	2	4	top 50 at time point 2 present at time point 1		
sample 7	15	30	top 50 at time point 1 present at time point 2		
+	2	4	top 50 at time point 1 present at top 50 time point 2		
	8	16	top 50 at time point 2 present at time point 1		
sample 8	32	64	top 50 at time point 1 present at time point 2		
-	7	14	top 50 at time point 1 present at top 50 time point 2		
	26	52	top 50 at time point 2 present at time point 1		
sample 9	11	22	top 50 at time point 1 present at time point 2		
+	7	14	top 50 at time point 1 present at top 50 time point 2		
	14	28	top 50 at time point 2 present at time point 1		
